# Supplementary material for: Poly(ADP-ribose) glycohydrolase coordinates meiotic DNA double-strand break induction and repair independent of its catalytic activity
Source: Nat Commun. 2020 Sep 25;11:4869. doi: 10.1038/s41467-020-18693-1 (PMC7519143; doi:10.1038/s41467-020-18693-1)
Supplement: Supplementary file 1 — Supplementary Information [file 41467_2020_18693_MOESM1_ESM.pdf]

**Supplementary Information**

# "Poly(ADP-ribose) glycohydrolase coordinates meiotic DNA double-strand break induction and repair independent of its catalytic activity"

Janisiw et al.

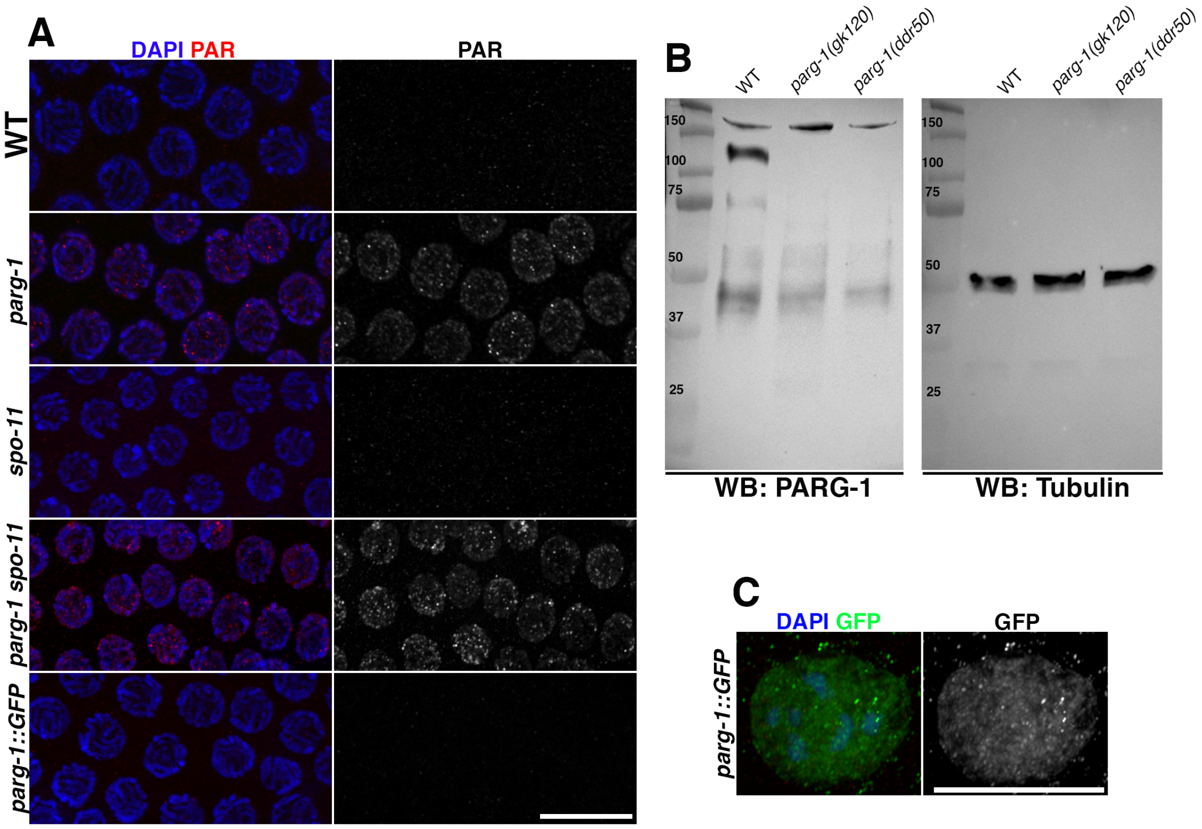


**Supplementary Figure 1. PAR accumulates in absence of endogenous DSBs. (A)** Immunostaining analysis showing accumulation of PAR in *parg-1* mutants even in absence of SPO-11-induced DSBs. *parg-1::GFP* does not accumulate PAR, indicating functionality of the fusion protein. Scale bar 10 μm. Analysis was performed in biological duplicates. **(B)** Western blot with anti-PARG-1 antibody on whole cell extract in WT, *parg-1(gk120)* and *parg-1(ddr50)*, showing that *parg-1* mutants are null. Tubulin was used as loading control. Analysis was performed in biological duplicates. **(C)** Representative image of a diakinesis nucleus from *parg-1::GFP* worms displaying mostly nucleoplasmic localization. Scale bar 5 μm. Analysis was performed in biological duplicates.

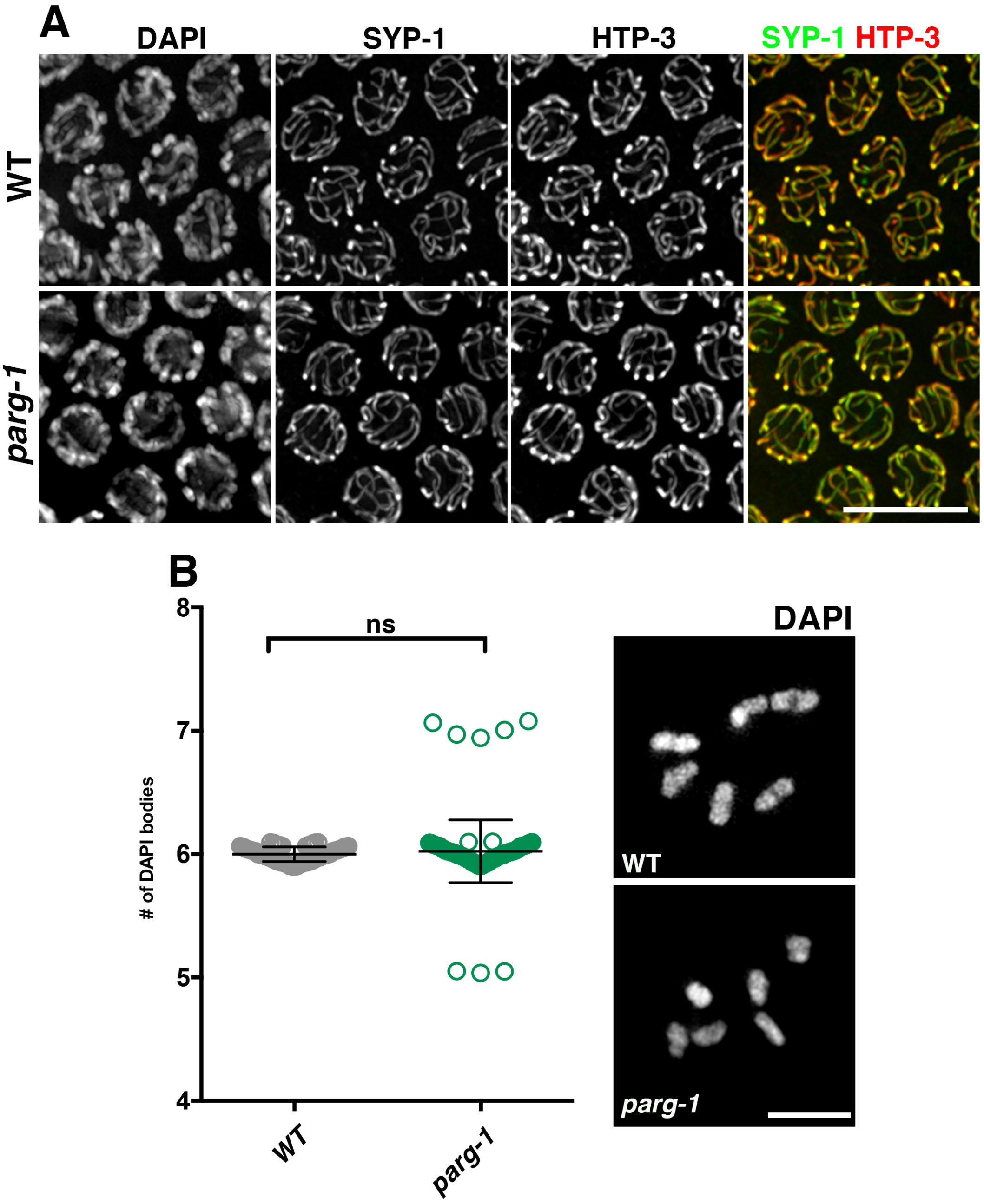


**Supplementary Figure 2. PARG-1 is dispensable for synapsis and CO formation. (A)** Staining of SYP-1 and HTP-3 shows no abnormalities in *parg-1(gk120)* mutants compared to wild-type (WT) animals. Analysis was performed in biological duplicates. **(B)** Quantification of DAPI-bodies (left) and representative examples (right) of diakinesis nuclei in the indicated genotypes. Scale bar 2 μm. Error bars indicate SD. Statistical analysis was conducted with two-tailed non-parametric Mann-Whitney test (*ns* indicates not significant difference). The number of nuclei analysed was WT (77) and *parg-1(gk120)* (128).

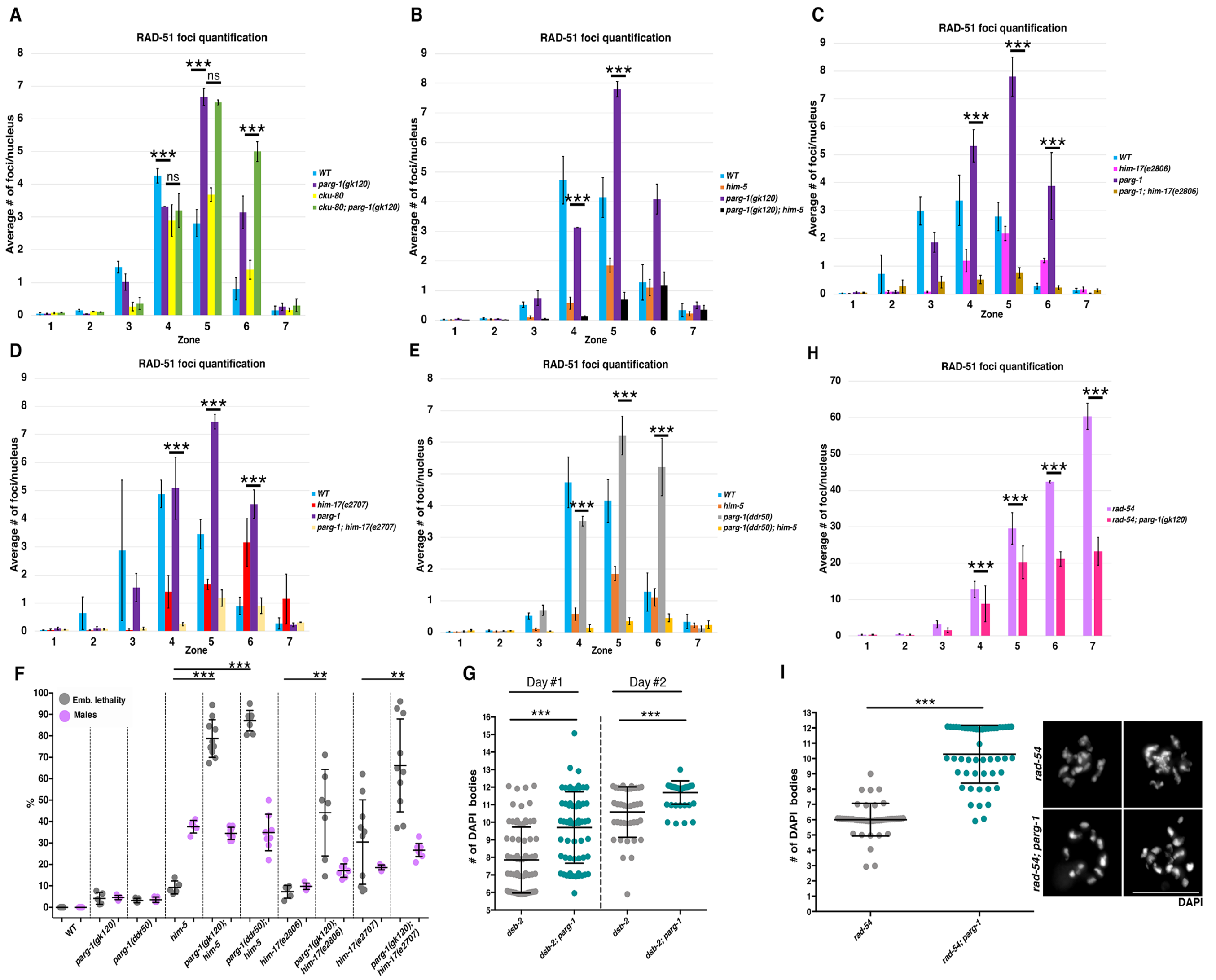


**Supplementary Figure 3. Abrogation of *parg-1* function causes reduced amounts of RAD-51 foci in mutants with reduced DSBs and *rad-54*-deficient worms. (A-E)** Quantification of RAD-51 foci throughout the germline in the indicated genotypes. Error bars indicate S.E.M. Statistical analysis was performed using non-parametric two-tailed Mann-Whitney test. (****p<0.0001*; *ns= not significant*). **(F)** Removal of *parg-1* causes synthetic lethality in DSB-defective mutants. Error bars indicate SD. (*him-5* vs *parg-1(gk120); him-5*, *p*= 0.0007 - *him-5* vs *parg-1(ddr50); him-5*, *p*= 0.00016 - *him-17(e2806)* vs *parg-1; him-17(e 2806)*, *p*= 0.0025 - *him-17(e2707)* vs *parg-1; him-17(e2707)*, *p*= 0.0015). **(G)** The CO defects in *dsb-2* mutants are exacerbated by lack of PARG-1. Error bars indicate SD.

Statistical analysis was conducted by non-parametric two-tailed Mann-Whitney test (***$p$<0.0001). **(H)** Abrogation of *parg-1* function reduces RAD-51 foci numbers in the *rad-54* mutant. Error bars indicate S.E.M. Statistical analysis was performed with non-parametric two-tailed Mann-Whitney test. (***$p$<0.0001). **(I)** Removal of *parg-1* in *rad-54* mutants triggers formation of univalents and largely alleviates chromosome morphology in diakinesis nuclei. Error bars indicate SD. Scale bar 5 μm. Statistical analysis was performed with non-parametric two-tailed Mann-Whitney test. (***$p$<0.0001).

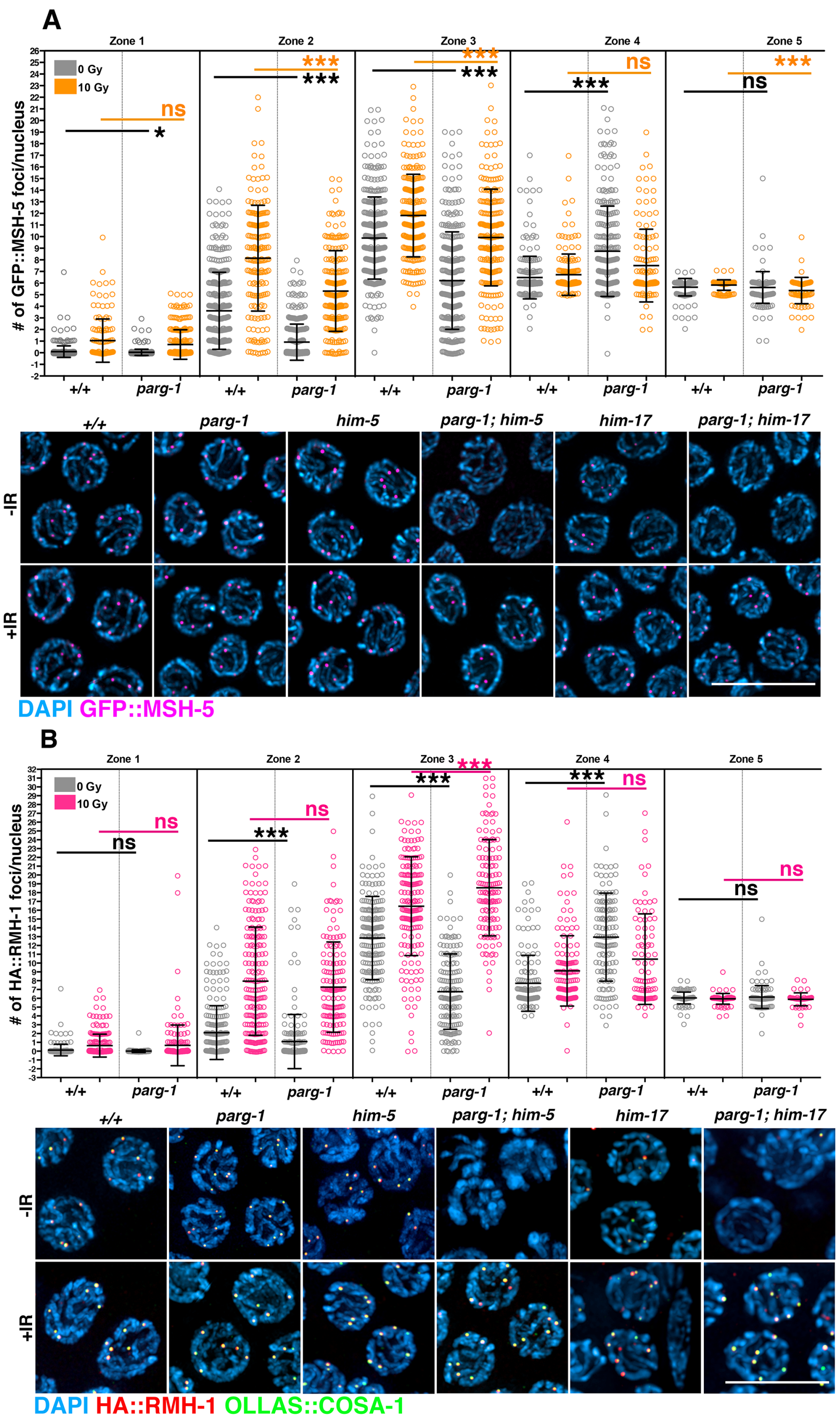


**Supplementary Figure 4. Loading of MSH-5 and RMH-1 is delayed in *parg-1* mutants and it is severely impaired in *parg-1; him-5* and *parg-1; him-17* double mutants. (A)** Top: quantification of GFP::MSH-5 foci number in controls and *parg-1* mutants. Note that *parg-1* mutants display a delayed accumulation of MSH-5. Bottom: late pachytene nuclei of

different genotypes stained for GFP (MSH-5) before and after IR. Scale bar 10 μm. Error bars indicate SD. **(B)** Top: quantification of HA::RMH-1 foci number in controls and *parg-1* mutants. Note that as for MSH-5, *parg-1* mutants display a delayed accumulation of RMH-1 as well. Bottom: late pachytene nuclei of different genotypes stained for HA (RMH-1) and OLLAS (COSA-1) before and after IR. Scale bar 10 μm. Error bars indicate SD. Statistical analysis was performed using non-parametric two-tailed Mann-Whitney test. (***$p<0.0001$; *$p< 0.01$; *ns= not significant*).

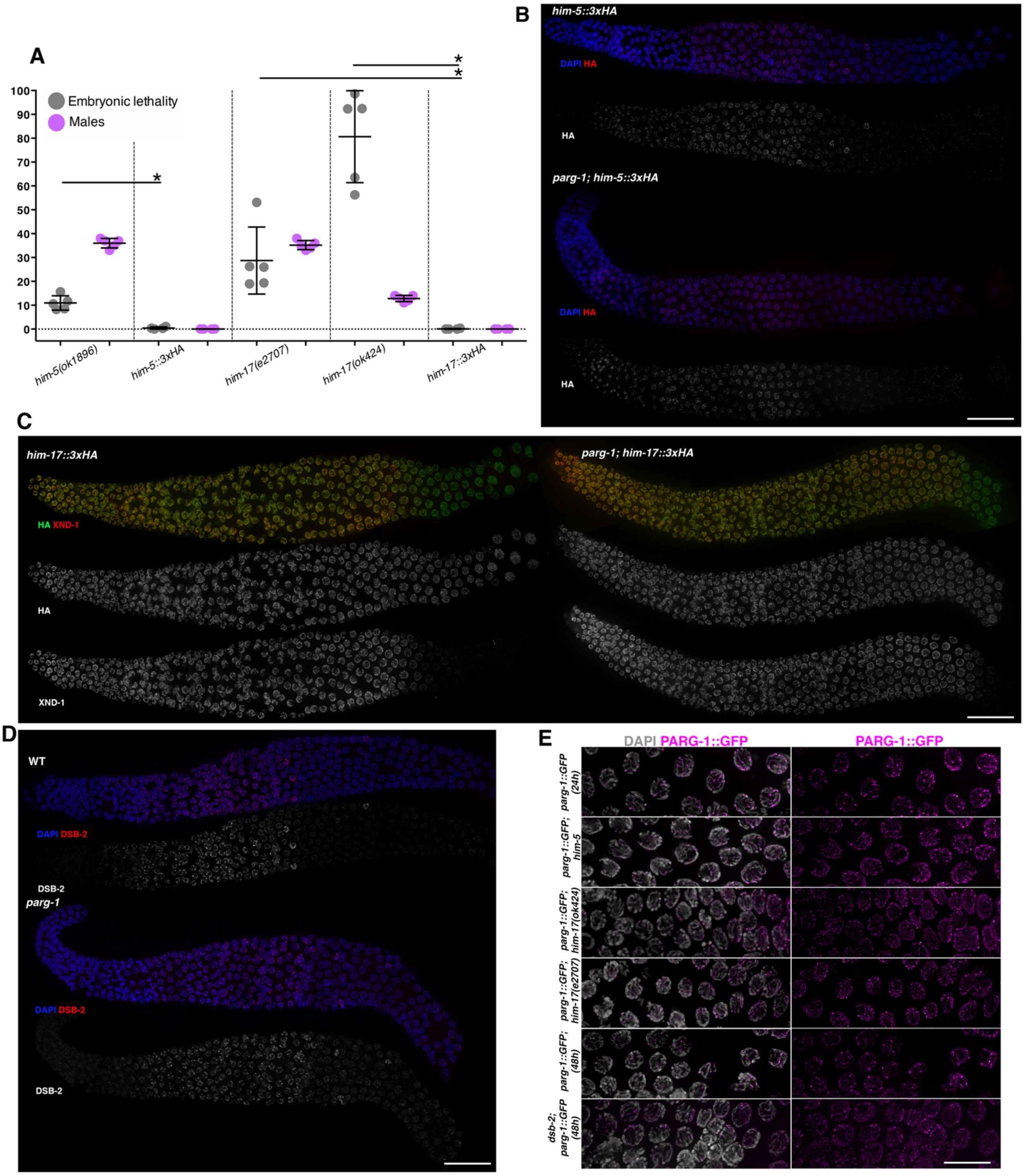


**Supplementary Figure 5. PARG-1 and pro-DSB factors display a mutually independent loading. (A)** Viability and male progeny assessment revealed full functionality of *him-5::3xHA* and *him-17::3xHA* tagged lines compared to respective mutant backgrounds. Error bars indicate SD. Statistical analysis was performed using non-parametric two-tailed

Mann-Whitney test. (*$p<0.01$). Number of worms scored: *him-5* (5), *him-5::3xHA* (4), *him-17(e2707)* (5), *him-17 (ok424)* (5), *him-17::3xHA* (4). **(B)** HIM-5 shows normal loading in *parg-1* mutant germlines. Scale bar 20 μm. Analysis was performed in biological duplicates. **(C)** HIM-17 and XND-1 do not display loading abnormalities in *parg-1* mutants. Scale bar 20 μm. Analysis was performed in biological duplicates. **(D)** *parg-1* is not required for DSB-2 loading. Scale bar 20 μm. Analysis was performed in biological duplicates. **(E)** Loading of PARG-1 is not dependent on *him-5*, *him-17* and *dsb-2*. Scale bar 20 μm. Analysis was performed in biological duplicates.

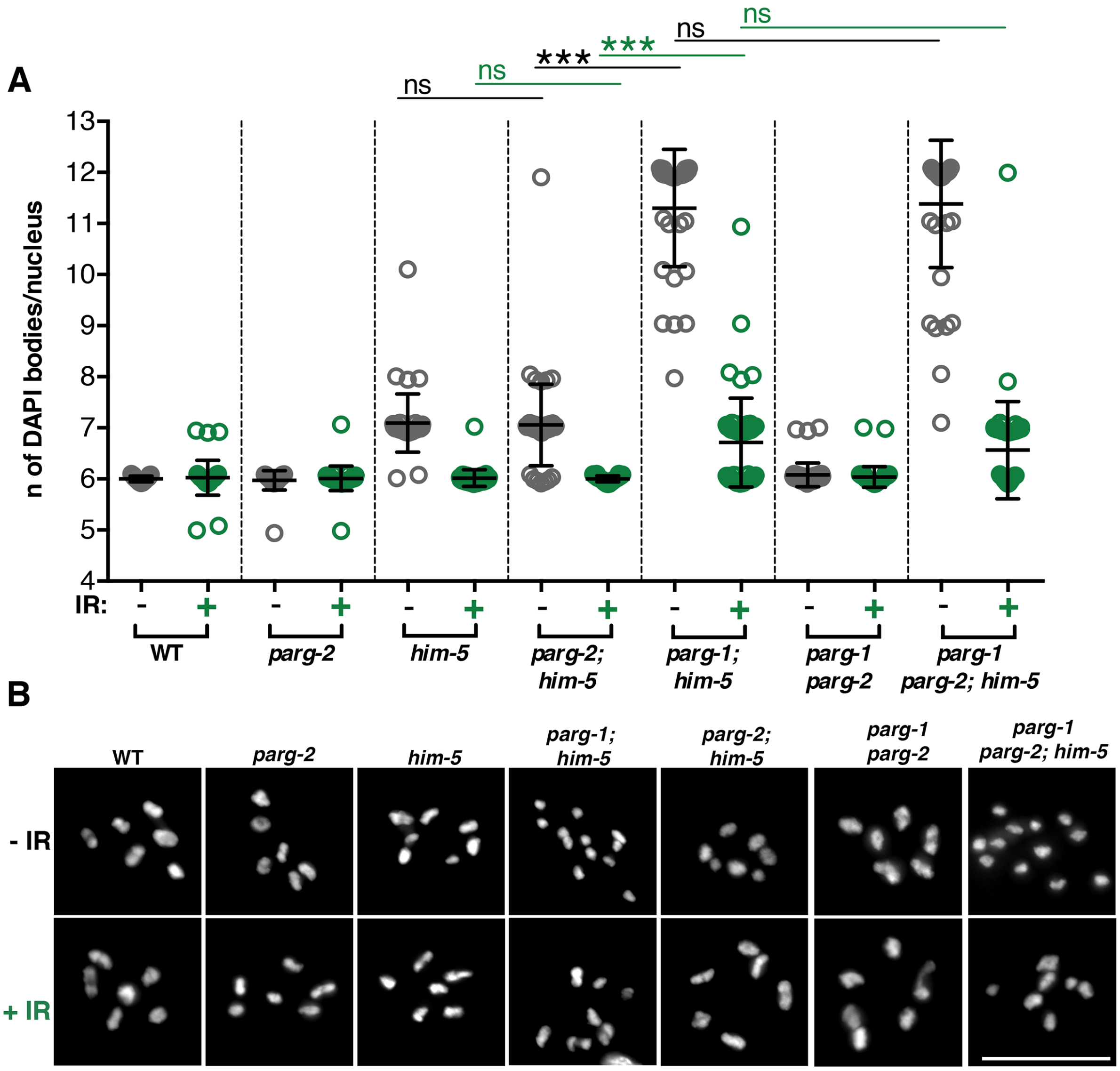


**Figure S6. Loss of *parg-2* does not cause synthetic phenotypes with *him-5* irrespectively of *parg-1*. (A)** Quantification of DAPI-bodies in diakinesis nuclei of the indicated genotypes before and after IR exposure. Error bars indicate SD. Statistical analysis was performed using non-parametric two-tailed Mann-Whitney test. (****p<0.0001*; *ns= not significant*). Number of nuclei scored is reported in Supplementary Table 2. **(B)** Representative images of diakinesis nuclei of the indicated genotypes stained with DAPI. Scale bar 5 μm.

A B

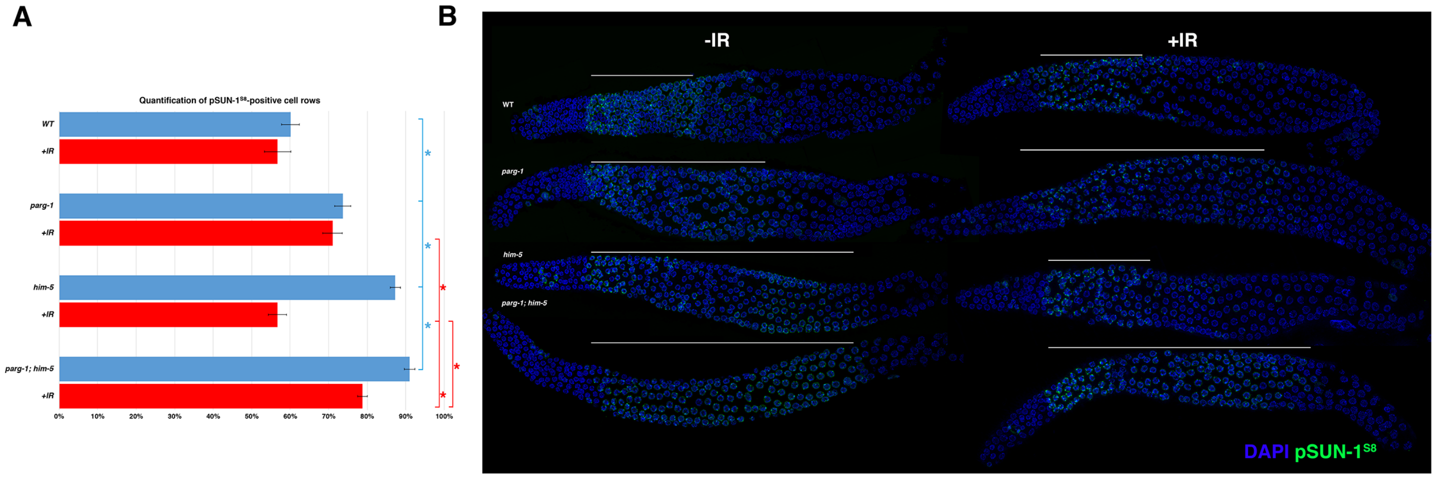


**Supplementary Figure 7. IR does not fully suppress accumulation of pSUN-1$^{S8}$ in *parg-1; him-5* double mutants. (A)** Quantification of pSUN-1$^{S8}$-positive nuclei cell rows in the indicated genotypes before and after IR exposure. Error bars indicate S.E.M. Statistical analysis was performed using unpaired *t* test. (*$p < 0.01$)**.** Number of gonads analysed (0 Gy-10Gy): WT (4-3), *parg-1* (4-4), *him-5* (3-5), *parg-1; him-5* (5-4). **(B)** Representative whole-mount gonads of the indicated genotypes before and after irradiation, stained for pSUN-1$^{S8}$ and DAPI. Scale bar 30 μm.

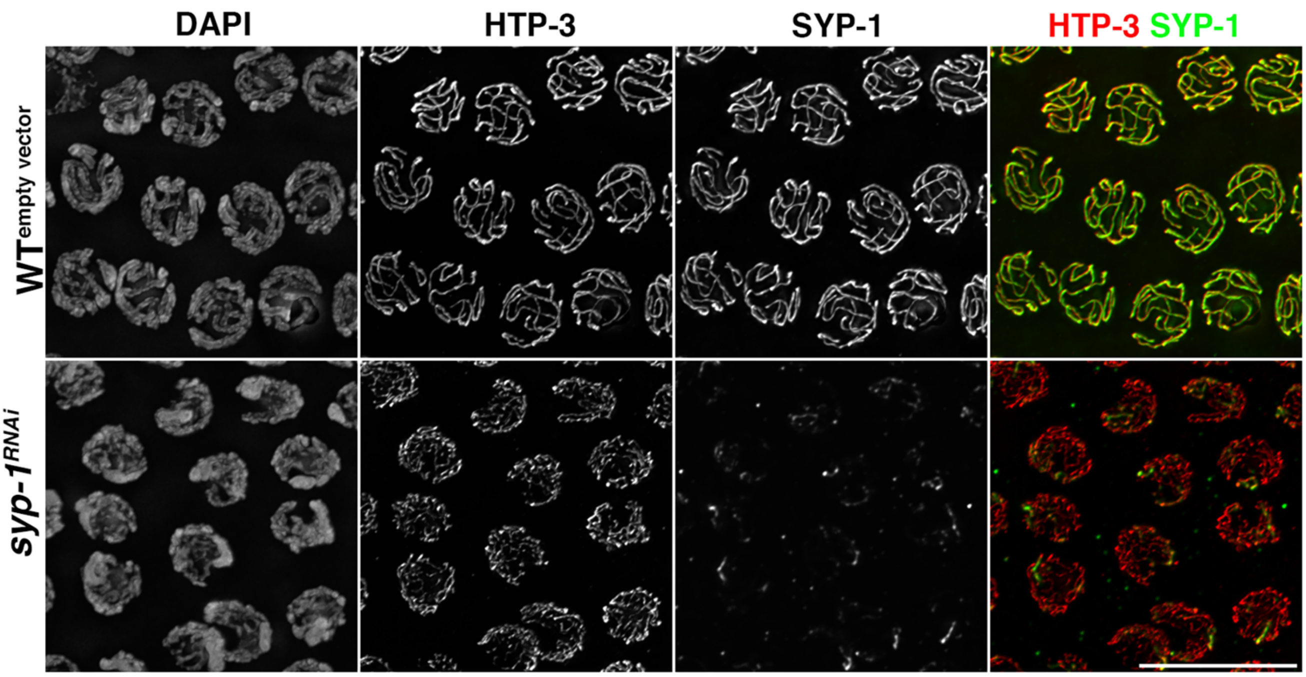


**Supplementary Figure 8. Rabbit anti-SYP-1 antibody is specific.** Immunostaining analysis in control worms (empty vector) and upon *syp-1RNAi* shows specific detection of SYP-1. Note that SYP-1 is robustly detected along the SC together with HTP-3 but the signal is dramatically reduced under (incomplete) *syp-1RNAi*. Scale bar 10 μm. Analysis was performed in biological duplicates.

**Supplementary Table 1.**

| Number of nuclei analysed for GFP::MSH-5 quantification | | | | | |
|---|---|---|---|---|---|
| Genotype | Zone 1 | Zone 2 | Zone 3 | Zone 4 | Zone 5 |
| *GFP::msh-5* | 179 | 142 | 159 | 117 | 72 |
| *GFP::msh-5 +IR* | 130 | 170 | 212 | 165 | 105 |
| *GFP::msh-5 parg-1* | 207 | 178 | 176 | 143 | 58 |
| *GFP::msh-5 parg-1 +IR* | 234 | 194 | 211 | 129 | 74 |
| *GFP::msh-5; him-5* | 276 | 239 | 205 | 170 | 107 |
| *GFP::msh-5; him-5 +IR* | 235 | 246 | 219 | 149 | 82 |
| *GFP::msh-5 parg-1; him-5* | 195 | 166 | 141 | 114 | 79 |
| *GFP::msh-5 parg-1; him-5 +IR* | 225 | 202 | 190 | 126 | 63 |
| *GFP::msh-5; him-17* | 379 | 359 | 268 | 215 | 131 |
| *GFP::msh-5; him-17 +IR* | 181 | 178 | 162 | 100 | 76 |
| *GFP::msh-5 parg-1; him-17* | 454 | 417 | 329 | 251 | 230 |
| *GFP::msh-5 parg-1; him-17 +IR* | 182 | 147 | 134 | 84 | 54 |
| **Number of nuclei analysed for HA::RMH-1 quantification** | | | | | |
| Genotype | Zone 1 | Zone 2 | Zone 3 | Zone 4 | Zone 5 |
| *HA::rmh-1* | 196 | 224 | 181 | 153 | 101 |
| *HA::rmh-1 +IR* | 204 | 195 | 171 | 134 | 79 |
| *HA::rmh-1; parg-1* | 209 | 198 | 148 | 130 | 106 |
| *HA::rmh-1; parg-1 +IR* | 174 | 141 | 131 | 87 | 60 |
| *HA::rmh-1; him-5* | 201 | 218 | 172 | 135 | 80 |
| *HA::rmh-1; him-5 +IR* | 201 | 123 | 118 | 78 | 50 |
| *HA::rmh-1; parg-1; him-5* | 209 | 168 | 136 | 89 | 78 |
| *HA::rmh-1; parg-1; him-5 +IR* | 226 | 175 | 141 | 102 | 59 |
| *HA::rmh-1; him-17* | 226 | 207 | 158 | 101 | 85 |
| *HA::rmh-1; him-17 +IR* | 162 | 144 | 96 | 86 | 68 |
| *HA::rmh-1; parg-1; him-17* | 189 | 155 | 138 | 99 | 92 |
| *HA::rmh-1; parg-1; him-17 +IR* | 234 | 215 | 155 | 97 | 75 |

**Supplementary Table 2.**

| Number of diakinesis nuclei analysed (from Fig. 4) | | |
|---|---|---|
| Genotype | 0 Gy | 10 Gy |
| WT | 75 | 28 |
| *parg-1(gk120)* | 127 | 43 |
| *parg-1(ddr50)* | 57 | 56 |
| *him-5* | 85 | 38 |
| *parg-1(gk120); him-5* | 92 | 44 |
| *parg-1(ddr50); him-5* | 51 | 62 |
| *him-17(e2806)* | 161 | 67 |
| *parg-1(gk120); him-17(e2806)* | 135 | 94 |
| *him-17(e2707)* | 105 | 52 |
| *parg-1(gk120); him-17(e2707)* | 119 | 35 |
| *spo-11* | 49 | 60 |
| *parg-1 spo-11* | 43 | 62 |
| **Number of diakinesis nuclei analysed (from Fig. 6)** | | |
| Genotype | n | - |
| WT | 40 | - |
| *parg-1(cd)* | 56 | - |
| *him-5* | 36 | - |
| *parg-1(cd); him-5* | 42 | - |
| *parg-1(gk120); him-5* | 44 | - |
| *parp-1; parg-1(gk120); him-5* | 33 | - |
| *parp-2; parg-1(gk120); him-5* | 35 | - |
| *parp-1; parp-2; parg-1(gk120); him-5* | 51 | - |
| **Number of diakinesis nuclei analysed (from Supp. Fig. 6)** | | |
| Genotype | 0 Gy | 10 Gy |
| WT | 33 | 40 |
| *parg-2* | 34 | 40 |
| *him-5* | 44 | 45 |
| *parg-2; him-5* | 57 | 53 |
| *parg-1; him-5* | 33 | 59 |
| *parg-1 parg-2* | 51 | 53 |
| *parg-1 parg-2; him-5* | 44 | 49 |

## Supplementary Table 3.

| Number of nuclei scored for quantification of OLLAS::COSA-1 foci | | | |
|---|---|---|---|
| Genotype | 0 Gy | 10 Gy (8h) | 10 Gy (17h) |
| *OLLAS::cosa-1* | 130 | 170 | 118 |
| *OLLAS::cosa-1; parg-1* | 119 | 101 | 102 |
| *OLLAS::cosa-1; him-5* | 109 | 128 | 156 |
| *OLLAS::cosa-1; parg-1; him-5* | 206 | 113 | 132 |
| **Number of nuclei scored for quantification of synapsis** | | | |
| Genotype | 0 Gy | 10 Gy (8h) | 10 Gy (17h) |
| *OLLAS::cosa-1* | 108 | 177 | 149 |
| *OLLAS::cosa-1; parg-1* | 116 | 112 | 125 |
| *OLLAS::cosa-1; him-5* | 116 | 140 | 184 |
| *OLLAS::cosa-1; parg-1; him-5* | 234 | 131 | 160 |

## Supplementary Table 4.

### Chromosome I

| ***Genetic Interval*** / ***Genotype*** | **AB** | **BC** | **CD** | **DE** | **EF** | **FG** | **GH** |
|---|---|---|---|---|---|---|---|
| **WT** | 22.54 (15) | 5.9 (4) | 13.4 (9) | 7.46 (5) | 11.94 (8) | 7.46 (5) | 31.43 (21) |
| ***parg-1(gk120)*** | 4.61** (3) | 3.07 (2) | 29.23** (23) | 21.53* (19) | 15.38 (12) | 3.07 (3) | 30.76 (27) |
| ***parg-1(cd)*** | 19.54 (17) | 8.04 (7) | 14.94 (13) | 2.29 (2) | 9.19 (9) | 5.74 (5) | 40.22* (35) |

The number of COs per interval is shown in brackets. The change in crossover distribution between *wild type* and *parg-1(gk120)* is statistically significant ($\chi^2$ = 51.845, *p< 0.001*). Crossover distribution is not significantly different between wild type and *parg-1(cd)* mutants ($\chi^2$= 6.83, *p= 0.3369*). *, ** refer to statistical significance in recombination frequency within the interval between *wild type* and *parg-1(gk120)* *(significance level for p<0.05, * p<0.05 - **p<0.001)*

### Chromosome V

| ***Genetic Interval*** / ***Genotype*** | **AB** | **BC** | **CD** | **DE** |
|---|---|---|---|---|
| **WT** | 35.48 (22) | 6.45(4) | 6.45 (4) | 51.61 (32) |
| ***parg-1(gk120)*** | 11.84** (19) | 15.41* (21) | 39.52** (51) | 33.54* (50) |
| ***parg-1(cd)*** | 28.78 (19) | 13.63 (9) | 6.06 (4) | 51.51 (34) |

The number of COs per interval is shown in brackets. The change in crossover distribution between *wild type* and *parg-1(gk120)* is statistically significant ($\chi^2$=234.97, *p< .001*). Crossover distribution is not significantly different between wild type and *parg-1(cd)* mutants ($\chi^2$= 6.0638, *p= 0.1085*). *, ** refer to statistical significance in recombination frequency within the interval between *wild type* and *parg-1(gk120)* *(significance level for p<0.05, * p<0.05 - **p<0.001)*

## Supplementary Table 5

| Number of nuclei scored for quantification of RAD-51 foci | | | | | | | |
|---|---|---|---|---|---|---|---|
| Genotype | Zone 1 | Zone 2 | Zone 3 | Zone 4 | Zone 5 | Zone 6 | Zone 7 |
| WT | 222 | 196 | 158 | 137 | 146 | 121 | 117 |
| *parg-1* | 130 | 170 | 122 | 114 | 105 | 79 | 93 |
| *spo-11* | 239 | 233 | 218 | 199 | 156 | 155 | 93 |
| *spo-11 parg-1* | 197 | 211 | 200 | 185 | 132 | 102 | 78 |
| *him-5* | 257 | 250 | 182 | 191 | 181 | 145 | 79 |
| *parg-1(gk120); him-5* | 180 | 220 | 177 | 172 | 103 | 153 | 97 |
| *him-17(e2806)* | 273 | 233 | 238 | 255 | 254 | 201 | 124 |
| *parg-1(gk120); him-17(e2806)* | 199 | 198 | 150 | 126 | 166 | 143 | 88 |
| *him-17(e2707)* | 204 | 197 | 171 | 174 | 128 | 78 | 55 |
| *parg-1(gk120); him-17(e2707)* | 172 | 189 | 154 | 135 | 107 | 80 | 78 |
| *parg-1(ddr50)* | 196 | 226 | 158 | 153 | 145 | 136 | 102 |
| *parg-1(ddr50); him-5* | 208 | 257 | 188 | 175 | 156 | 115 | 88 |
| *rad-54* | 137 | 167 | 109 | 97 | 99 | 87 | 83 |
| *rad-54; parg-1* | 129 | 181 | 157 | 107 | 90 | 72 | 69 |
| *cku-80* | 136 | 254 | 219 | 208 | 184 | 166 | 149 |
| *cku-80; parg-1(gk120)* | 207 | 277 | 206 | 181 | 141 | 116 | 116 |

## Supplementary Table 6.

| Genotype | Source | Id. number |
|---|---|---|
| *parp-1(ddr31) I.* | This study | NSV167 |
| *rmh-1(jf172[HA::rmh-1]) I.* | This study | NSV240 |
| *htp-3::TEV::eGFP::myc::3xFLAG I.* | CGC | JH4008 |
| *rad-54(tm1268)/hT2[bli-4(e937) let-?(q782) qIs48] (I;III)* | Jantsch lab | NSV187 |
| *parp-2(ok344) II.* | CGC | VC1171 |
| *dsb-2(me96) II.* | Villeneuve lab | AV477 |
| *GFP::cosa-1 II.* | Villeneuve lab | AV630 |
| *cosa-1(tm3298)/qC1[dpy-19(e1259) glp-1(q339) qIs26] III.* | Villeneuve lab | AV590 |
| *oxTi574 II; unc-119(ed3) III.* | CGC | EG7875 |
| *cosa-1(ddr12[OLLAS::cosa-1]) III.* | Janisiw et al.; 2018 | NSV97 |
| *parg-1(gk120) IV.* | CGC | VC130 |
| *parg-1(ddr50) IV.* | This study | NSV277 |
| *parg-2(ddr20) IV.* | This study | NSV140 |
| *parg-1(ddr3[parg-1::GFP]) IV.* | This study | NSV25 |
| *parg-1(gk120) parg-2(ddr20) IV.* | This study | NSV126 |
| *msh-5(ddr22[GFP::msh-5]) IV* | Janisiw et al.; 2018 | NSV129 |
| *parg-1(ddr29[parg-1$^{E554,555A}$::GFP]) IV.* | This study | NSV162 |
| *parg-1(ddr34[parg-1$^{E554,555A}$]) IV.* | This study | NSV177 |
| *parg-1$^{gk120}$(ddr51) IV in CB4856.* | This study | NSV278 |
| *him-5(ok1896) V.* | CGC | RB1562 |
| *him-17(e2806) V.* | This study | NSV169 |
| *him-17(e2707) V.* | This study | NSV170 |

| | | |
|---|---|---|
| *him-17(ddr37[him-17::3xHA]) V.* | This study | NSV205 |
| *syp-2(ok307)7nT1 [unc-?(n754) let-?(m435)] (IV;V).* | CGC | AV276 |
| *parp-2 (ok344) II; parg-1(gk120) IV.* | This study | NSV09 |
| *spo-11(ok79)/nT1 [unc-?(n754) let-?] (IV;V).* | CGC | AV106 |
| *syp-3(ok758) I; ieSi11 II; unc-119(ed3) III.* | CGC | CA1218 |
| *parg-1(gk120) IV; him-5(ok1896) V.* | This study | NSV56 |
| *parg-1(gk120) spo-11(ok79)/nT1 [unc-?(n754) l et-?] (IV;V).* | This study | NSV74 |
| *parg-1(gk120) with Hawaiian chromosome V* | This study | NSV121 |
| *parg-1(ddr3[parg-1::GFP]) IV; him-5(ok1896) V.* | This study | NSV124 |
| *cosa-1(ddr12[OLLAS::cosa-1]) III; parg- 1(gk120) IV.* | This study | NSV137 |
| *cosa-1(ddr12[OLLAS::cosa-1]) III; him- 5(ok1896) V.* | This study | NSV138 |
| *cosa-1(ddr12[OLLAS::cosa-1]) III; parg- 1(gk120) IV; him-5(ok1896) V.* | This study | NSV139 |
| *cku-80(ok861) III.* | CGC | RB964 |
| *cosa-1(ddr12[OLLAS::cosa-1]) III; parg- 1(gk120); him-5(ok1896)/nT1[unc-?(n754) let-?] (IV* | This study | NSV155 |
| *parg-1(gk120) msh-5(ddr22[GFP::msh-5]) IV* | This study | NSV157 |
| *parg-1(ddr3[parg-1::GFP]) IV; syp- 2(ok307)7nT1 [unc-?(n754) let-?(m435)] (IV;V).* | This study | NSV160 |
| *dsb-2(me96) II; parg-1(gk120) IV.* | Yanowitz lab | QP1367 |
| *mre-11(iow1)/nT1 [qIs51] (IV;V).* | CGC | SSM2 |
| *parg-1(gk120) IV; him-17(e2806) V.* | Yanowitz lab | QP1374 |
| *parg-1(gk120) IV; mre-11(iow1)/nT1 [qIs51] (IV;V).* | Yanowitz lab | QP1377 |
| *parp-1(ddr31) I; parp-2(ok344) II.* | This study | NSV175 |
| *parg-1(ddr34[parg-1$^{E554,555A}$]) IV in CB4856.* | This study | NSV178 |
| *rmh-1(jf54)/ hT2 [bli-4(e937) let-?(q782) qIs48] (I;III).* | This study | NSV180 |
| *parg-1(ddr34[parg-1$^{E554,555A}$::GFP]) IV; him- 5(ok1896) V.* | This study | NSV181 |
| *com-1(t1626) unc-32(e189)/hT2 [bli-4(e937) let- ?(q782) qIs48] (I;III).* | This study | NSV182 |
| *parp-1(ddr31) I; parg-1(gk120) IV.* | This study | NSV184 |
| *cosa-1(tm3298)/qC1[dpy-19(e1259) glp- 1(q339) qIs26] III; parg-1(ddr3[parg-1::GFP]) IV.* | This study | NSV186 |
| *parg-1(ddr34[parg-1$^{E554,555A}$]) IV; him-5(ok1896) V.* | This study | NSV190 |
| *com-1(t1626) unc-32(e189)/hT2 [bli-4(e937) let- ?(q782) qIs48] (I;III); parg-1(gk120) IV.* | This study | NSV194 |
| *him-17(ok424)/nT1 [unc-?(n754) let-?] (IV;V).* | This study | NSV196 |
| *parp-1(ddr31) I; parp-2(ok344) II; parg-1(gk120) IV.* | This study | NSV199 |
| *parg-1(gk120) IV; him-17(ddr37[him-17::3xHA] V.* | This study | NSV206 |
| *parg-1(gk120)/nT1 [unc-?(n754) let-?] (IV;V).; him-17(ok424)/nT1 [unc-?(n754) let-?] (IV;V).* | This study | NSV209 |

| | | |
|---|---|---|
| *parg-1(gk120)/nT1 [unc-?(n754) let-?] (IV;V).; him-17(e2707)/nT1 [unc-?(n754) let-?] (IV;V).* | This study | NSV236 |
| *msh-5(ddr22[GFP::msh-5]) IV; him-5(ok1896) V.* | This study | NSV241 |
| *msh-5(ddr22[GFP::msh-5]) IV; him-17(e2707) V.* | This study | NSV242 |
| *parp-2(ok344) II; parg-1(gk120) IV; him- 5(ok1896) V.* | This study | NSV244 |
| *him-17(e2707)/nT1 [unc-?(n754) let-?] (IV;V).* | This study | NSV246 |
| *msh-5(ddr22[GFP::msh-5])/nT1 [unc-?(n754) l et-?] (IV;V); him-17(e2707)/nT1 [unc-?(n754) let-?] (IV;V).* | This study | NSV247 |
| *him-5(ddr43[him-5::3xHA]) V.* | This study | NSV250 |
| *parg-1(gk120) IV; him-5(ddr43[him-5::3xHA]) V.* | This study | NSV252 |
| *rmh-1(jf172[HA::rmh-1]); cosa- 1(ddr12[OLLAS::cosa-1]) III; parg-1(gk120) IV.* | This study | NSV253 |
| *parg-1(gk120) msh-5(ddr22[GFP::msh-5])/nT1 [unc-?(n754) let-?] (IV;V); him-17(e2707)/nT1 [unc-?(n754) let-?] (IV;V).* | This study | NSV254 |
| *htp-3(tm3655)/hT2 [bli-4(e937) let-?(q782) qIs48] (I;III).* | This study | NSV255 |
| *rmh-1(jf172[HA::rmh-1]); cosa- 1(ddr12[OLLAS::cosa-1]) III; him-5(ok1896) V.* | This study | NSV256 |
| *rmh-1(jf172[HA::rmh-1]); cosa-1(ddr12[OLLAS::cosa-1]) III; parg-1(gk120) IV; him- 5(ok1896) V.* | This study | NSV257 |
| *rmh-1(jf172[HA::rmh-1]) I; cosa- 1(ddr12[OLLAS::cosa-1]) III.* | This study | NSV258 |
| *parg-1(ddr3[parg-1::GFP]) IV; him-5(ddr43[him- 5::3xHA]) V.* | This study | NSV262 |
| *htp-3(tm3655)/hT2 [bli-4(e937) let-?(q782) qIs48] (I;III); parg-1(ddr3[parg-1::GFP]) IV.* | This study | NSV265 |
| *parg-1(ddr3[parg-1::GFP]) IV; him- 17(ddr37[him-17::3xHA]) V.* | This study | NSV267 |
| *parg-1(gk120) msh-5(ddr22[GFP::msh-5]) IV; him-5(ok1896) V.* | This study | NSV273 |
| *rmh-1(jf172[HA::rmh-1])I; cosa- 1(ddr12[OLLAS::cosa-1]) III; him-17(e2707) V.* | This study | NSV276 |
| *dsb-2(me96) II; parg-1(ddr3[parg-1::GFP]) IV.* | This study | NSV280 |
| *parg-1(ddr3[parg-1::GFP]) IV; him-17(e2707) V.* | This study | NSV281 |
| *rmh-1(jf172[HA::rmh-1]) I; cosa- 1(ddr12[OLLAS::cosa-1]) III; parg-1(gk120)/nT1 [unc- ?(n754) let-?] (IV;V); him-17(e2707)/nT1 [unc-?(n754) let-?] (IV;V).* | This study | NSV286 |
| *cosa-1(ddr12[OLLAS::cosa-1]) III; parg- 1(ddr3[parg-1::GFP]) IV.* | This study | NSV292 |
| *parg-1(ddr3[parg-1::GFP])/nT1 [unc-?(n754) l et-?] (IV;V); him-17(ok424)/nT1 [unc-?(n754) let-?] (IV;V).* | This study | NSV294 |

**List of primers used in the study.**

| Primers Name | Sequences | Amplicone |
|---|---|---|
| PR185<br>PR186 | 5´-CCGTCACGACAAAATGCAGA-3´<br>5´-TGCCACCTTCTCCAATGACT-3´ | *parp-1(ddr31)*<br>*Deletion only* |
| PR301<br>PR302 | 5´-ACAGAGTACCACTTCACCTTTCC-3´<br>5´-AATGCAACTTTTACTGCGCTCG-3´ | *HA::rmh-1* |
| PR305<br>PR306 | 5´-GCCTGCCATAGAACCACCTG-3´<br>5´-GGCGTCGCGACCTCTAGAAA-3´ | *rad-54(tm1268)* |
| PR011<br>PR012 | 5´-CGAGAAAACTCATCTTCATTG-3´<br>5´-AAAGGATTAGCAACACCTTCAGA-3´ | *parp-2(ok344)* |
| PR100<br>PR101 | 5´-CAATGTCAAGTTCTCGGTGA-3´<br>5´-AAAATTTGCGAAAAATGGTC-3´ | *cosa-1(tm3298)* |
| PR55<br>PR56 | 5´-ACTTTGTATTGGTCTCGCAC-3´<br>5´-AACCTGATTCGCTGCTGATA-3´ | *OLLAS::cosa-1* |
| PR005<br>PR006 | 5´-ATTTTGACAAGGCGAGAGGA-3´<br>5´-CGAGCTTACGTTTGATGCTT-3´ | *parg-1(gk120)*<br>*deletion only* |
| PR021<br>PR022 | 5´-GATTGCATTTACTGAGGGAAA-3´<br>5´-CATGATTTTCAACTGTGACCA-3´ | *parg-1(gk120)*<br>*N2 only* |
| PR027<br>PR028 | 5´-AAATGTAGAGCGAGATTTTG-3´<br>5´-TGAAATGGTCATGATCAGTT-3´ | *parg-2(ddr20)* |
| PR205<br>PR206 | 5´-TGCTAACTTACAGGCATGCT-3´<br>5´-TGCATGGATTGGTTTCACAC-3´ | *parg-1::GFP* |
| PR33<br>PR34 | 5´-GTTTTTCAGCAACCTTTTCTT-3´<br>5´-ATTATCATCACGGGGTAAGG-3´ | *GFP::msh-5* |
| PR154<br>PR155 | 5´-TGTGGAGAAACTTAAATTCCTGT-3´<br>5´-TCGCTTCCAGTTGTTTCATTT-3´ | *parg-1(cd)*<br>*digested with*<br>*AluI* |
| PR220<br>PR221 | 5´-CGGAGCTCAAAATGAAAAGAGCAGC-3´<br>5´-CGATGCGACCAACTGTTTTTCG-3´ | *him-5(ok1896)* |
| PR199<br>PR200 | 5´-TGGAGGGAGAAGAAGAAGAAGT-3´<br>5´-TCAGAAACAACGCAACGAATT-3´ | *him-17::3xHA* |
| PR45<br>PR46 | 5´-AGAGTCAGCTTCGCATCTCC-3´<br>5´-AAAAGGAAACTACGCCATCG-3´ | *syp-2(ok307)* |
| PR75<br>PR76 | 5´-CGTGGAAACATTGCTTGAAA-3´<br>5´-GGACTGATGGAACCGAGAAA-3´ | *spo-11(ok79)* |
| PR203<br>PR204 | 5´-TTCGATGCCATACACCTTCA-3´<br>5´-TCAAAGTCATCCGAATCTATTGG-3´ | *him-17(ok424)* |
| PR102<br>PR103 | 5´-GACTGTTTTCCAGGCGTAAA-3´<br>5´-AATTACCCTTTTTGGGTTCA-3´ | *htp-3(tm3655)* |
| PR228<br>PR229 | 5´-TGAACAGTCACAGGAAATCAC-3´<br>5´-AGTTATATGCCCGAGTGTCC-3´ | *him-5::3xHA* |
| I, -19 | 5´-ATGCCAGTGATAAGGAACGG-3´<br>5´-TCACATCCCTTGTCGATGAA-3´ | snp_F56C11 |
| I, -12 | 5´-TCGAAATCAGGGAAAAATTGA-3´<br>5´-ACGATTTTCGGGGAGTTTTT-3´ | snp_Y71G12 |
| I, -6 | 5´-GTTTTCACTTTTGCCGGTGT-3´<br>5´-TGAAGGCGCATATACAGCAG-3´ | pkP1052 |
| I, -1 | 5´-AAAATATCAGGAAAGAGTTTCGG-3´<br>5´-TTTAAAGATTAAGGGTGGAGCG-3´ | snp_D1007 |
| I, 5 | 5´-ATCTGGCACCAAATATGAGTCG-3´<br>5´-AATCTCGATTTTCAAGGAGTGG-3´ | CE1-247 |
| I, 13 | 5´-TCCTGGATAATCCCCAAAAA-3´ | snp_F58D5 |

| | | |
|---|---|---|
| | 5´-CCCTGCCATTGATCTTGTTT-3´ | |
| I, 14 | 5´-TTGAAATCCCCTTTAAAATCCC-3´<br>5´-ACACTGGGTACCTGACTCATGC-3´ | uCE1-1361 |
| I, 26 | 5´-ATTATTAACGGCCACGGTGA-3´<br>5´-CCCACACACTCTCACCTTCA-3´ | snp_Y105E8B |
| V, -17 | 5´-TTTCGGAAAATTGCGACTGT-3´<br>5´-CGCGTTTTGGAGAATTGTTT-3´ | pkP5076 |
| V, -5 | 5´-GAGATTCTAGAGAAATGGACACCC-3´<br>5´-AAAAATCGACTACACCACTTTTAGC-3´ | snp_Y61A9L |
| V, 1 | 5´-AGAAATGATCCGATGAAAAAGC-3´<br>5´-CCGATAGTGTTCATAGCATCCC-3´ | pkP5097 |
| V, 6 | 5´-CAAATTAAATATTTCTCAAAGTTTCGG-3´<br>5´-ACATAAGCGCCATAACAAGTCG-3´ | R10D12 |
| V, 18 | 5´-GAAATTCAAATTTTTGAGAAACCC-3´<br>5´-TTCAGACCATTTTTAGAATATTCAGG-3´ | snp_Y17D7B |

## Uncropped blots from Figure 2

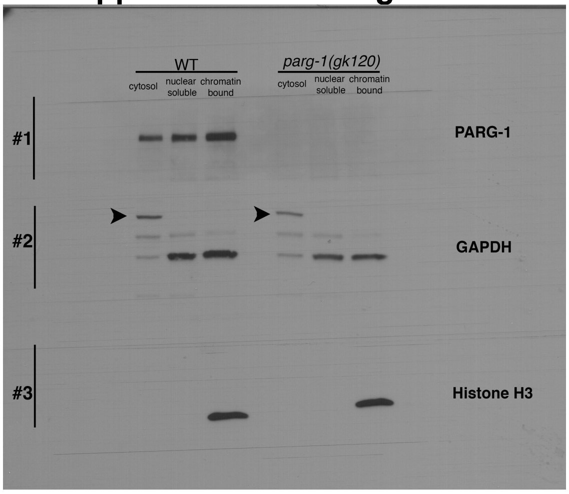


Fig. 2a

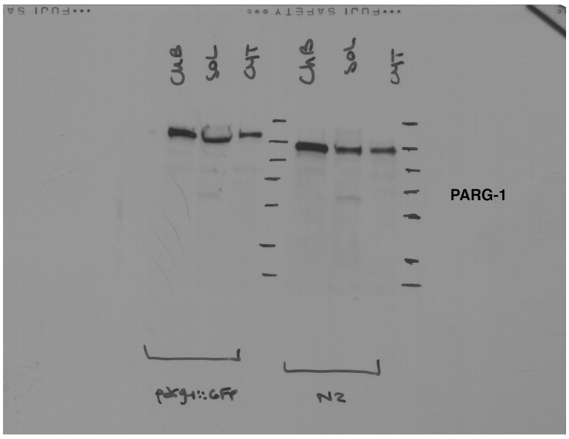


Fig. 2b

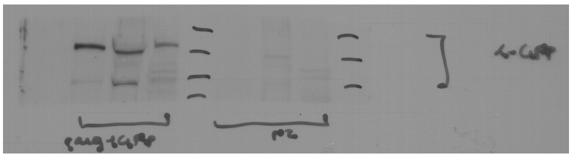

Fig. 2b

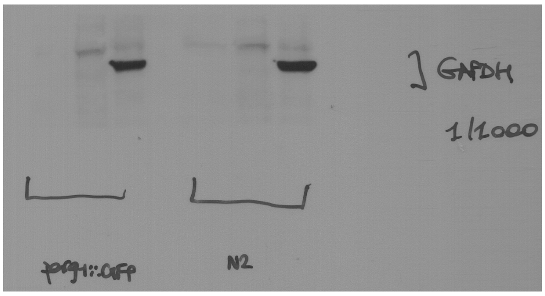


Fig. 2b

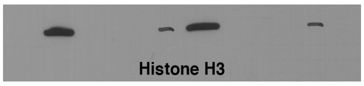


Fig. 2b
