# Supplementary material for: Poly(ADP-ribose) glycohydrolase coordinates meiotic DNA double-strand break induction and repair independent of its catalytic activity
Source: Nat Commun. 2020 Sep 25;11:4869. doi: 10.1038/s41467-020-18693-1 (PMC7519143; doi:10.1038/s41467-020-18693-1)
Supplement: Supplementary file 3 — Reporting Summary [file 41467_2020_18693_MOESM3_ESM.pdf]

nature research

Corresponding author(s): Nicola Silva, PhD

Last updated by author(s): August 26, 2020

Reporting Summary

Nature Research wishes to improve the reproducibility of the work that we publish. This form provides structure for consistency and transparency in reporting. For further information on Nature Research policies, see our Editorial Policies and the Editorial Policy Checklist.

Statistics

For all statistical analyses, confirm that the following items are present in the figure legend, table legend, main text, or Methods section.

n/a	Confirmed	
☐	☒	The exact sample size (*n*) for each experimental group/condition, given as a discrete number and unit of measurement
☐	☒	A statement on whether measurements were taken from distinct samples or whether the same sample was measured repeatedly
☐	☒	The statistical test(s) used AND whether they are one- or two-sided<br>*Only common tests should be described solely by name; describe more complex techniques in the Methods section.*
☐	☒	A description of all covariates tested
☐	☒	A description of any assumptions or corrections, such as tests of normality and adjustment for multiple comparisons
☐	☒	A full description of the statistical parameters including central tendency (e.g. means) or other basic estimates (e.g. regression coefficient) AND variation (e.g. standard deviation) or associated estimates of uncertainty (e.g. confidence intervals)
☐	☒	For null hypothesis testing, the test statistic (e.g. *F*, *t*, *r*) with confidence intervals, effect sizes, degrees of freedom and *P* value noted<br>*Give P values as exact values whenever suitable.*
☒	☐	For Bayesian analysis, information on the choice of priors and Markov chain Monte Carlo settings
☒	☐	For hierarchical and complex designs, identification of the appropriate level for tests and full reporting of outcomes
☒	☐	Estimates of effect sizes (e.g. Cohen's *d*, Pearson's *r*), indicating how they were calculated

Our web collection on statistics for biologists contains articles on many of the points above.

Software and code

Policy information about availability of computer code

Data collection	Softworx software 6.5.2., used for image acquisition with Deltavision microscope system.<br>ZEN Blue 3.0, used for image acquisition with AxioImager microscope system.
Data analysis	Photoshop, Microsoft Excel, Prism, ImageJ.

For manuscripts utilizing custom algorithms or software that are central to the research but not yet described in published literature, software must be made available to editors and reviewers. We strongly encourage code deposition in a community repository (e.g. GitHub). See the Nature Research guidelines for submitting code & software for further information.

Data

Policy information about availability of data

All manuscripts must include a data availability statement. This statement should provide the following information, where applicable:

- Accession codes, unique identifiers, or web links for publicly available datasets
- A list of figures that have associated raw data
- A description of any restrictions on data availability

All data generated or analysed during this study are included in this published article (and its supplementary information files). The source data underlying Figs. 1B, D, 3A, C, 4B-D, 5A, B, F, G, 6F, G, I and Supplementary Figs. 2B, 3A-I, 4A, B, 5A, 6A and 7A are provided in Source Data-1 and Source Data-2 files respectively.

Field-specific reporting

Please select the one below that is the best fit for your research. If you are not sure, read the appropriate sections before making your selection.

☒ Life sciences ☐ Behavioural & social sciences ☐ Ecological, evolutionary & environmental sciences

For a reference copy of the document with all sections, see nature.com/documents/nr-reporting-summary-flat.pdf

Life sciences study design

All studies must disclose on these points even when the disclosure is negative.

Sample size	Sample sizes were chosen accordingly to common practice in the C. elegans meiosis field, where at least three gonads for each genotype are analyzed and the number of nuclei used for quantifications range between 40-100.
Data exclusions	No data was excluded from the analysis.
Replication	All the data presented in the manuscript were produced from successful biological replicates or triplicates.
Randomization	Samples were analyzed according to genotypes, and age-matched depending on the stage of meiotic prophase in which the analysis of the phenotypes was conducted (pachytene or diakinesis stages).
Blinding	Blinding was not relevant to the study, as animals needed to be preselected for age matching and therefore each of the genotypes needed to be identified.

Reporting for specific materials, systems and methods

We require information from authors about some types of materials, experimental systems and methods used in many studies. Here, indicate whether each material, system or method listed is relevant to your study. If you are not sure if a list item applies to your research, read the appropriate section before selecting a response.

Materials & experimental systems

n/a	Involved in the study	
☐	☒	Antibodies
☒	☐	Eukaryotic cell lines
☒	☐	Palaeontology and archaeology
☐	☒	Animals and other organisms
☒	☐	Human research participants
☒	☐	Clinical data
☒	☐	Dual use research of concern

Methods

n/a	Involved in the study	
☒	☐	ChIP-seq
☒	☐	Flow cytometry
☒	☐	MRI-based neuroimaging

Antibodies

Antibodies used

Rabbit polyclonal anti HA, Sigma H6908
Rabbit polyclonal anti OLLAS, Genscript A01658
Rabbit polyclonal anti PAR, Trevigen 4336-BPC-100
Mouse monoclonal anti GFP, Roche 11814460001
Guinea pig polyclonal anti HTP-3, (Goodyer et al., 2008)
Guinea pig polyclonal anti HTP-3, (Y. Kim lab)
Chicken polyclonal anti SYP-1, (Silva et al., 2014)
Rabbit polyclonal anti SYP-1, (This study)
Rabbit polyclonal anti HTP-1, (Martinez-Perez et al., 2008)
Rabbit polyclonal anti RAD-51, Novus 29480002
Guinea pig polyclonal anti phospho-SUN-1S8, (Woglar et al., 2013)
Rabbit polyclonal anti DSB-2, (Rosu et al., 2013)
Guinea pig polyclonal anti XND-1, (Wagner et al., 2010)
Mouse monoclonal anti H3K4me2, Millipore 05-1338
Mouse monoclonal anti HA, Cell Signalling 2367S
Mouse monoclonal anti PARG-1, Clone 2D4 (This study)
Chicken polyclonal anti GFP, Abcam ab13970
Rabbit polyclonal anti Histone H3, Abcam ab1791
Goat polyclonal anti actin, Santa Cruz Sc-1615
Mouse monoclonal anti Tubulin, Thermofisher T9026-100UL

	Mouse monoclonal anti GAPDH, Ambion AM4300<br>Goat anti Rabbit HRP-conjugated, ThermoFisher G21234<br>Goat anti Mouse HRP-conjugated, ThermoFisher G21040<br>Goat anti Chicken HRP-conjugated, ThermoFisher A16054
Validation	Validation of anti PARG-1 antibody was performed by Western Blot analysis, in which an immunoreactive band of the expected molecular weight was observed in wildtype but not in parg-1 mutant worms. Validation of anti SYP-1 antibody was performed by immunostaining on WT and syp-1RNAi animals, in which the known localization pattern of SYP-1 was observed in the former but not in the latter. All the remaining antibodies were either commercially available or already tested in previous studies. The antibodies directed against GFP, FLAG, HA and OLLAS tags were tested on the relative tagged lines, as well as in the untagged N2 wild type worms to assess whether any non-specific cross-reaction was present.

Animals and other organisms

Policy information about studies involving animals; ARRIVE guidelines recommended for reporting animal research

Laboratory animals	All research employed Caenorhabditis elegans nematodes and no vertebrate model systems were employed.
Wild animals	No wild animals were employed.
Field-collected samples	No animals collected from the field were employed.
Ethics oversight	For use of the invertebrate Caenorhabditis elegans, no ethical statement is required.

Note that full information on the approval of the study protocol must also be provided in the manuscript.
